# Mal-Prec: computational prediction of protein Malonylation sites via machine learning based feature integration: Malonylation site prediction

**DOI:** 10.1186/s12864-020-07166-w

**Published:** 2020-11-23

**Authors:** Xin Liu, Liang Wang, Jian Li, Junfeng Hu, Xiao Zhang

**Affiliations:** 1 Department of Bioinformatics, School of Medical Informatics and Engineering, Xuzhou Medical University, Xuzhou, 221004 Jiangsu China; 2 Jiangsu Key Laboratory of New Drug Research and Clinical Pharmacy, School of Pharmacy, Xuzhou Medical University, Xuzhou, 221000 Jiangsu China; 3 School of Public Health and Tropical Medicine, Tulane University, New Orleans, LA 70118 USA

**Keywords:** Post-translational modification, Malonylation, Machine learning, Principal component analysis, Support vector machine

## Abstract

**Background:**

Malonylation is a recently discovered post-translational modification that is associated with a variety of diseases such as Type 2 Diabetes Mellitus and different types of cancers. Compared with experimental identification of malonylation sites, computational method is a time-effective process with comparatively low costs.

**Results:**

In this study, we proposed a novel computational model called Mal-Prec (Malonylation Prediction) for malonylation site prediction through the combination of Principal Component Analysis and Support Vector Machine. One-hot encoding, physio-chemical properties, and composition of k-spaced acid pairs were initially performed to extract sequence features. PCA was then applied to select optimal feature subsets while SVM was adopted to predict malonylation sites. Five-fold cross-validation results showed that Mal-Prec can achieve better prediction performance compared with other approaches. AUC (area under the receiver operating characteristic curves) analysis achieved 96.47 and 90.72% on 5-fold cross-validation of independent data sets, respectively.

**Conclusion:**

Mal-Prec is a computationally reliable method for identifying malonylation sites in protein sequences. It outperforms existing prediction tools and can serve as a useful tool for identifying and discovering novel malonylation sites in human proteins. Mal-Prec is coded in MATLAB and is publicly available at https://github.com/flyinsky6/Mal-Prec, together with the data sets used in this study.

**Supplementary Information:**

The online version contains supplementary material available at 10.1186/s12864-020-07166-w.

## Background

Post-translational modification (PTM) participates in many biological processes through protein function regulations. It has been well recognized that PTM identification is critical in the prevention and medical treatment of certain diseases. Lysine malonylation (Kmal) is a novel type of PTMs that was initially detected by mass spectrometry and is widely present in both eukaryotic and prokaryotic organisms [1]. For instance, Kmal has been enriched in key signaling molecules in mouse liver [2], plant cells [3] and the gram-positive bacterium *Saccharopolyspora spinosa*, etc. [4, 5]. Although many efforts have been devoted to investigating the cellular mechanisms of Kmal, its biological significance remains poorly understood [2, 6]. Recognition of malonylation sites in substrates represents an initial but crucial step in elucidating the molecular mechanisms underlying protein malonylation. With the development of high-throughput mass spectrometry techniques, many Kmal-containing peptides have been identified [7, 8]. However, considering the dynamic properties and low abundance of malonylation and the limitation of experiment methods, identification of the exact substrates or sites on a large scale remains challenging.

To date, various computational tools have been developed to predict malonylation sites in protein sequences [9–14]. For instance, Xu et al. [9] used minimum Redundancy Maximum Relevance (mMRM) model to construct a prediction tool named Mal-Lys by incorporating residue sequence order information, position-specific amino acid propensity, and physicochemical properties for each peptide. Wang et al. [10] built a predictor called MaloPred, which took into accounts of five features including amino acid compositions (AAC), amino acids binary encoding (BINA), encoding based on grouped weight (EBGW), K nearest neighbors feature (KNN), and position specific scoring matrix (PSSM). Their information gains (IG) were then evaluated to select most meaningful and significant features. Hasan and Kurata [11] proposed a prediction tool called identification of Lysine-Malonylation Sites (iLMS), which used the composition of profile-based k-Spaced Amino Acid Pairs (pkSAAP), dipeptide amino acid compositions (DC) and amino acid index properties (AAindex) to encode the segment. Chen et al. [12] constructed a LSTM-based ensemble malonylation predictor (LEMP), which combined the long short-term memory (LSTM) algorithm with word embedding and the random forest algorithm with novel encoding of enhanced amino acid content. In addition, Taherzadeh et al. [13] developed the SPRINT-Mal tool and found that evolutionary information and physicochemical properties are the two most discriminative features. A structural feature called half-sphere exposure provides additional improvement to the prediction performance. Bao et al. [14] proposed the IMKPse model that utilized general PseAAC as the classification features and employed flexible neural tree as classification model. Although many achievements have been made in the prediction of malonyl acylation modification sites, there is still much room for improvement in the prediction performance.

In this study, we investigated whether dimensionality reduction algorithm PCA is useful for predicting malonylation sites. Another issue that we attempted to address here is whether the integration of sequence features could generate better prediction accuracy. On the basis of our results, Mal-Prec significantly outperformed existing predictors and indicated that PCA, together with three sequence features, one-hot encoding, physiochemical properties (AAindex), and composition of k-spaced amino acid pairs (CKSAAP), is able to improve the accuracy of prediction. Thus, Mal-Prec could serve as a powerful tool for identifying malonylation sites in proteins.

## Results and discussion

### Determination of CKSAAP features

Though many approaches have adopted CKSAAP features to predict PTM sites, most of them only used the CKSAAP features generated by single K value and did not identify optimal K for constructing the CKSAAP feature. In order to obtain valid CKSAAP features, we analyzed the performance of different combination of CKSAAP features. In particular, we not only analyzed the CKSAAP features obtained by single K value ranging from 0 to 6, but also analyzed their combined effects. All data sets were dimensioned to 100 using PCA. We used LIBSVM tool which is available on https://www.csie.ntu.edu.tw/~cjlin/libsvmtools/. By using the grid search method, we optimized the two important parameters of SVM, c and g, which are the penalty parameters and kernel parameters respectively in the SVM algorithm. Finally, we set c = 10 and g = 2 in the SVM model and the radial basis function was adopted as the kernel function. 5-fold cross-validation was executed for 50 times to optimize the parameters in the training model. The results are shown in Supplementary Table 1, according to which, Acc, F1, and MCC do not change much under different K value. For example, the Sen value changes from 81.46 to 98.63%, the Spec value changes from 65.72 to 82.24%. Thus, it is difficult to figure out which is more suitable.

**Fig. 1:**
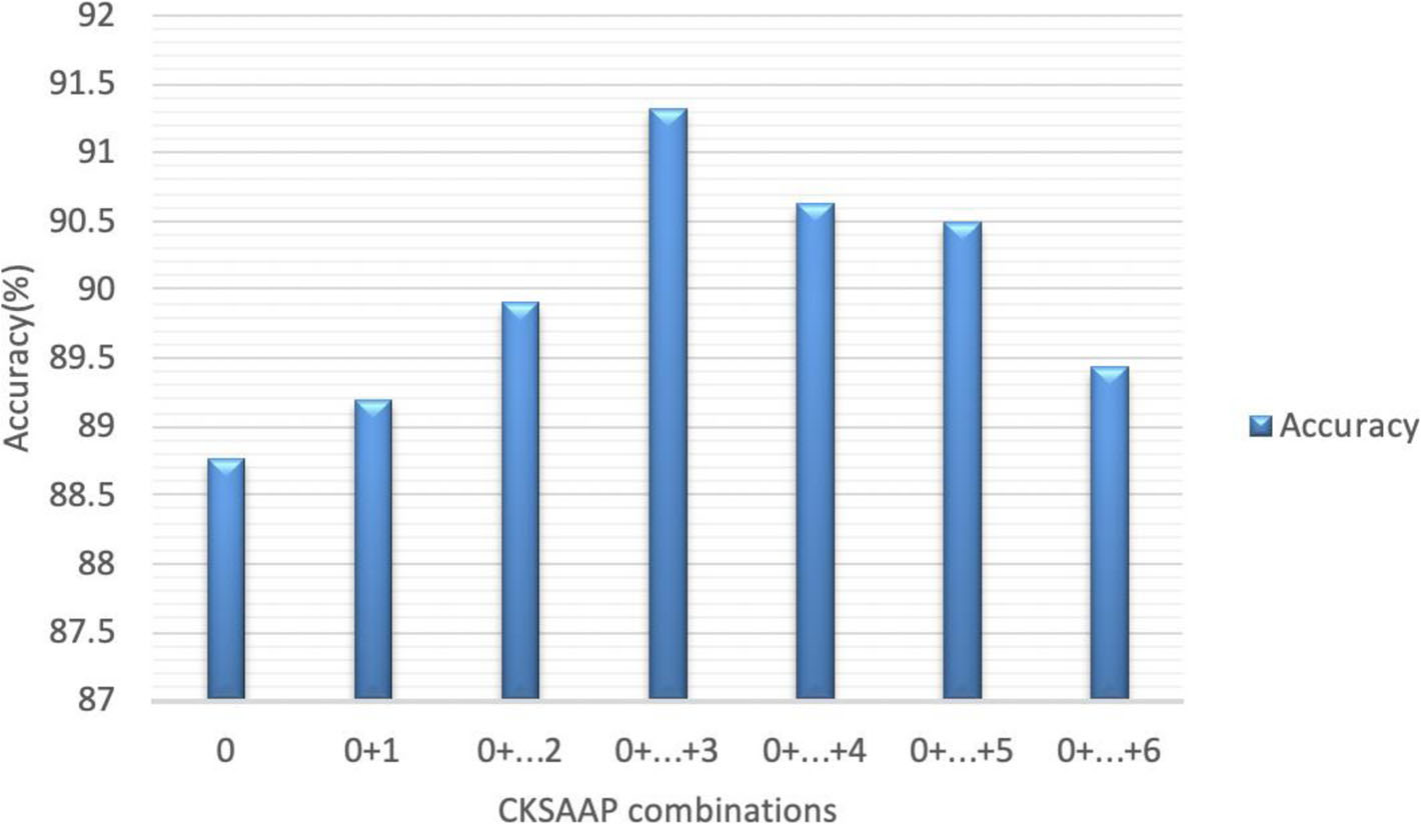
Comparison of accuracy of different CKSAAP feature combinations

Thereafter, we made comparisons by combining all features together (CKSAAP, one-hot encoding, AAindex). The parameters c and g in SVM were set to 1.9 and 0.07 by grid search, respectively. The performance is shown in Supplementary Table 2, according to which, we can see that when K was set to 0 to 6, the performance of the proposed method did not change too much. Acc, Sen, Spec, F1, and MCC changed from 88.58 to 89.87%, 89.01 to 90.38%, 87.53 to 89.77%, 88.65 to 89.87%, and 79.79 to 81.81%, separately. When combing feature vectors computed by different K value, the result has a certain law, which is shown in Fig. 1, from which we could see that when we combined the first 4 CKSSAP features together, the accuracy achieves the best score, so do in the other four metrics. Thus, in this paper, we set K as 0,1,2, and 3, and got the CKSAAP feature vectors were 441*4 = 1764-dimensions.

### Effectiveness of PCA

In order to determine the suitable dimensions of PCA for our prediction, we run the training model when the dimensions equal to 50, 100, 150, 200, 250, and 300, separately. 5-fold cross-validation was executed for 50 times to optimize the parameters. The results are shown in Table 1.

**Table 1:** 5-fold cross-validation results of different dimensions

dimensions	Acc (%)	Sen (%)	Spec (%)	F1 (%)	MCC (%)
50	85.91	86.09	85.68	85.94	75.77
100	**91.24**	**91.71**	**90.83**	**91.18**	**84.03**
150	90.20	91.00	89.48	90.30	82.31
200	88.11	89.53	86.65	88.39	79.02
250	84.61	86.15	83.10	84.73	73.91
300	82.31	84.34	80.36	82.27	70.89

In Table 1, when the dimensions equal to 100, the proposed method performed best, and average ACC, Sen, Spec, F1, and MCC can reach to 91.24, 91.71, 90.83, 91.18, and 84.03%, separately. Supplementary Figure 1 shows the accuracy curve in different dimensions. It is apparent that accuracy curve is a convex function. When the dimensions are equal to 100, the accuracy reaches the maximum of 91.24%. When the dimension value is greater than 100, larger the dimension gets, lower the accuracy is.

**Fig. 2:**
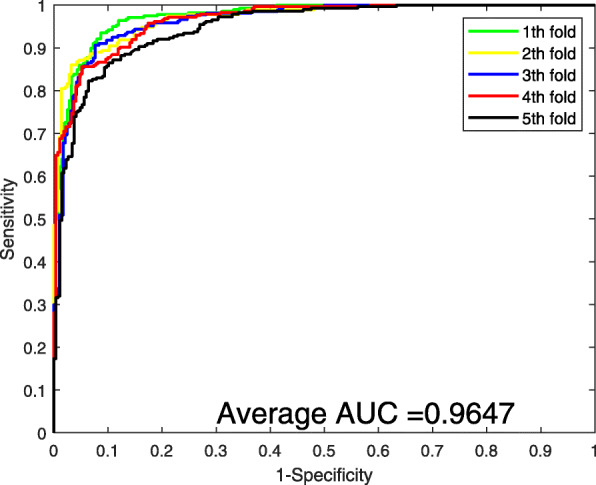
ROC curves of 5-fold cross-validation performed by SVM (dimensions equal to 100)

Supplementary Table 3 shows the performance of 5-fold cross-validation when implementing the proposed method on human data set. It can be seen that the average Acc, Sen, Spec, F1, and MCC can reach 91.24, 91.71, 90.83, 91.18, and 84.03%, separately. The standard deviations of these criteria values are 1.24, 2.50, 2.10, 1.43, and 2.09%, respectively. The ROC curves of the 5-fold cross-validation are listed in Fig. 2. The average AUC value is 96.47%.

**Fig. 3:**
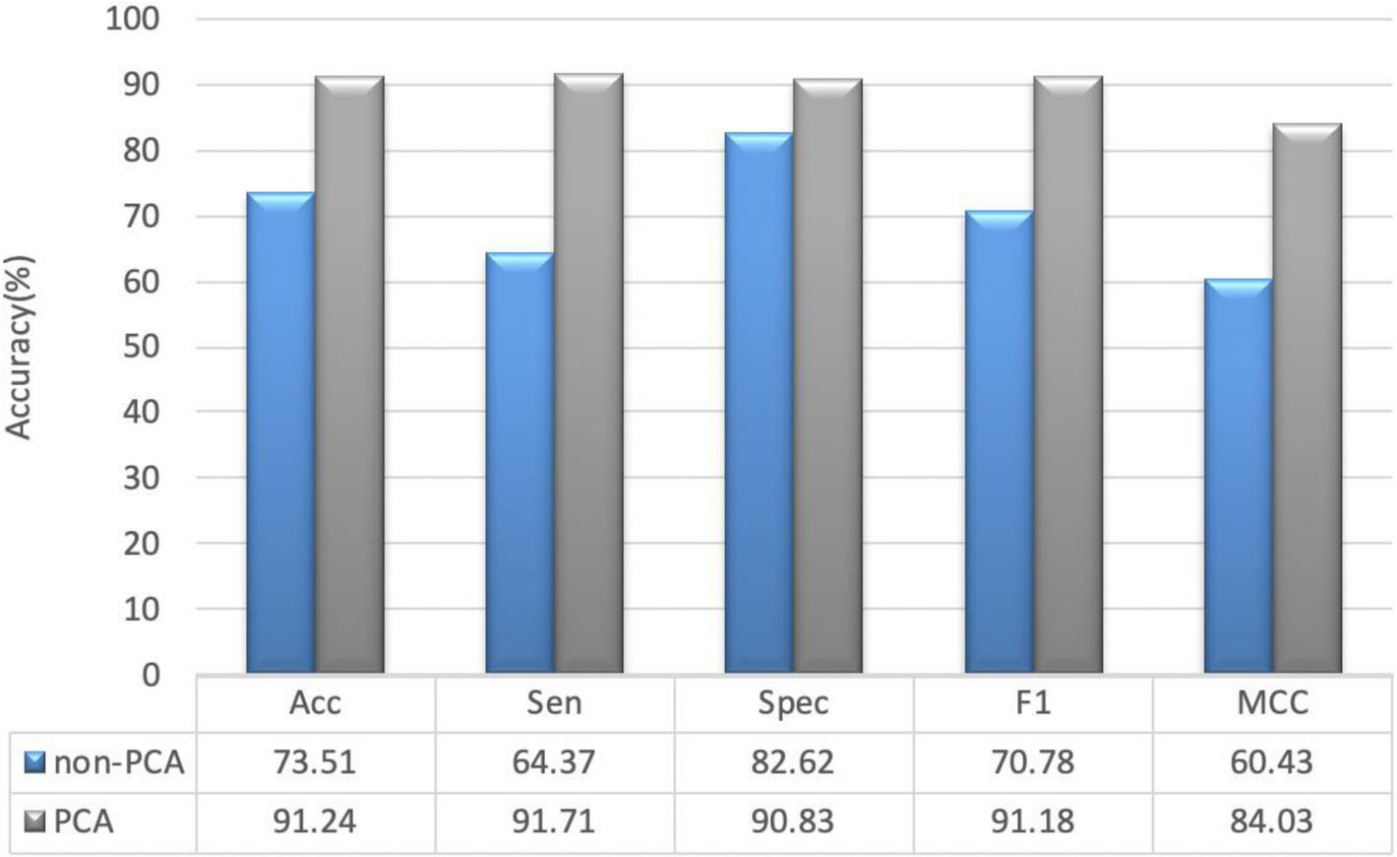
The comparison of different metrics result using PCA or not

For the purpose of analyzing the role of PCA in our proposed method, we applied the same procession of our proposed approach without PCA. The parameter c and g were set to 2 and 0.1 by grid search. The performance of the 5-fold cross-validation is shown in Supplementary Table 3, in which, the average Acc, Sen, Spec, F1, and MCC reach to 73.51, 64.37, 82.62, 70.78, and 60.43%. And the standard deviations of these criteria values are 1.99, 2.32, 2.63, 2.64, and 1.89%, respectively. For a more intuitive analysis, we adopted Fig. 3 to show the comparison of different metrics result using PCA or not. Non-PCA represents PCA was not used, PCA represents the dimensions are reduced to 100 using PCA. From Fig. 3 we could see that, by comparing to the proposed method without PCA, the average Acc, Sen, Spec, F1, and MCC of the proposed method with PCA could increase 17.73, 27.34, 8.21, 20.4, and 19.6%, respectively. That means PCA can effectively improve the performance of the algorithm.

### Performance comparison of different feature combination

For the purpose of further identifying the role of various features, we analyzed the performance of each feature and multiple feature combinations. The performance comparison of each single feature was shown in Supplementary Table 4, from which we could see that the CKSAAP outperforms the other two features, especially in terms of ACC, Sen, Spec, and F1, which are almost 20% ~ 30% higher than the other two features. Meanwhile, while the performance comparison of multiple features was shown in Supplementary Table 5, which shows the performance of different features combination. The CKSAAP (exclude) means exclude the CKSAAP from the three features, so it represents the combination of AAindex and One-hot. The AAindex (exclude) and One-hot (exclude) also has the same meaning. All represents the combination of three features. From Supplementary Table 6 we could see that the combination of AAindex and One-hot performs best in all of those two features combined. It is interesting because we know CKSAAP performs best in the comparison of a single feature. Thus, we can use a Chinese saying to summarize this phenomenon, three cobblers combined makes a genius mind. This is to say, the combination of the three features works best. For a more intuitive analysis, we applied the column chart to show the performance comparison of the seven kinds of feature combination. In Supplementary Figure 2, the ECKSAAP means exclude the CKSAAP from the three features. The AAindex and One-hot also have similar meaning. It can be seen that the proposed method which combined all features achieves best in all metrics. The ECKSAAP ranks second in terms of Acc, Spec, F1, and MCC.

According to the above analysis, after combining the four attributes of CKSAAP, one-hot encoding and nine attributes of AAindex, and then using PCA to reduce the dimension to 100, Mal-Prec can achieve better performance.

### Comparison of classical algorithms

We also compared Mal-Prec with other four classical classifiers on the training data sets, including Random Forest (RF), K-nearest neighbors (KNN), Ensemble of decision tree and Naive Bayes (NB) [15–17]. The Euclidean distance was used in KNN algorithm, and the number of its neighbor is 2. The number of decision trees in RF and Ensemble was 20 and 50, separately. 5-fold cross-validation was conducted 50 times to each of them. The performance comparisons are shown in Table 2.

**Table 2:** The performance comparisons of different classical classifiers

classifier	Acc (%)	Sen (%)	Spec (%)	F1 (%)	MCC (%)
KNN	59.68	26.73	92.18	34.34	34.98
NB	83.24	84.17	82.36	83.39	72.11
RF	68.25	62.41	74.04	66.27	56.36
Ensemble	64.11	60.11	68.20	62.50	53.69
Mal-Prec (SVM)	**91.24**	**91.71**	**90.83**	**91.18**	**84.03**

Even though it is well known that the ensemble classifier is more accurate and robust than individual classifiers, it can be seen from Table 2 that, compared with other classical classifiers, Mal-Prec model performs best in all metrics. That means different data set requires different models.

### Performance on independent data set

For objective performance comparison, the independent data set which is truly blind to the training data set was adopted to evaluate the performance of the proposed method. As seen in Table 3, the proposed method performs best, including Acc, Sen, Spec, F1, and MCC values of 90.65, 89.71, 91.59, 90.62, and 83.04%, respectively.

**Table 3:** Performance of different feature combinations on the independent data set

Features	Acc (%)	Sen (%)	Spec (%)	F1 (%)	MCC (%)
CKSAAP	77.55	77.14	77.97	77.59	65.18
AAindex	61.73	65.43	57.97	63.26	52.61
One-hot	58.71	61.43	55.94	59.97	51.44
CKSAAP (exclude)	86.19	86.86	85.51	86.36	76.19
AAindex (exclude)	79.42	80.57	78.26	79.77	67.30
One-hot (exclude)	71.08	76.29	65.80	72.65	58.65
ALL	90.65	89.71	91.59	90.62	83.04

**Fig. 4:**
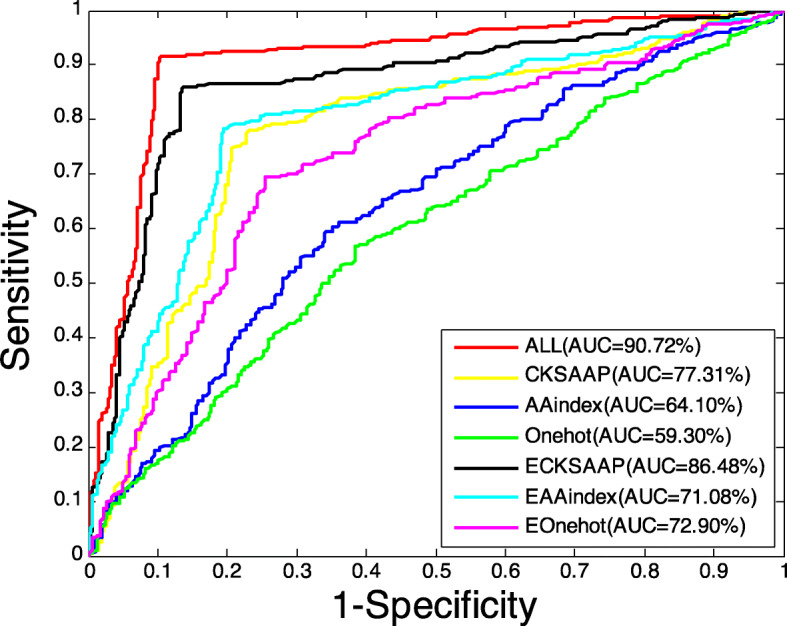
ROC curves performed by different feature combinations on the independent data set

Figure 4 shows the ROC curves from combinations of different features on the independent data set. It can be seen that, on the independent data set, the proposed method (all features) has a AUC value of 90.72%, the ECKSAAP ranks second, and the rest are ECKSAAP, EOne-hot, EAAindex, AAindex, One-hot, which are the same as the result on the testing data set. This further confirms that Mal-Prec constructed by incorporating those three features and PCA has a good effect.

### Comparison of the state-of-the-art approaches

We compared the proposed method with some state-of-the-art approaches for predicting malonylation sites, including Mal-Lys, MaloPred, iLMS, LEMP, SPRINT-Mal. Table 4 shows the comparison of the proposed method and some state-of-the-art approaches.

**Table 4:** Comparison of state-of-the-art approaches in terms of Acc and AUC in different organisms

Approach	Feature	Species	Acc (%)	AUC (%)
mRMR+SVM [9]	K-gram+AAindex	N/A	N/A	79.35
IG + SVM [10]	AAC + BINA (sequence-based)	*E. coli*	72.30	75.50
	EBGW (physicochemical)	Mouse	74.65	82.70
	KNN + PSSM (evolutionary)	*Homo sapiens*	73.72	87.10
IG + SVM [11]	PKsaap+AAindex+DC	Mouse	N/A	73.90
		*Homo sapiens*	N/A	74.30
LSTM+RF [12]	EAAC+word embedding	Mouse	88.00	82.40
		*Homo sapiens*		
SVM [13]	Binary+PSSM+AAindex+Structured (ASA + SS + HSE + IDR)	Mouse	N/A	76.00
Proposed method	AAindex+One-hot+CKSAAP	*Homo sapiens*	**90.65**	**90.91**

The reasons for the good performance of our proposed method can be summarized as two points. Firstly, PCA is utilized to extract features. PCA is a dimensionality reduction method, which extracts more effective characteristic information. Secondly, the support vector machine classifier is used for classification. All the above proves that the SVM classifier combined with principal component analysis and three features (PseAAC, One-hot, CKSAAP) is more suitable for predicting the malonylation sites than the state-of-the–art approaches.

### Feature analysis

**Fig. 5:**
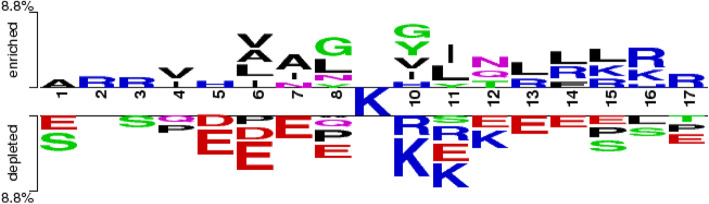
The statistical two-sample logo with t-test on human datasets (*P*-value < 0.05)

We also analyzed sequence occurrence frequency on every position using Two Sample Logo with t-test (*P*-value < 0.05). Figure 5 shows that the malonylation and non-malonylation peptides have considerably different sequence preferences. Glycine (G), Leucine (L), Alanine (A), and Valine (V) were significantly richer than those in non-malonylation ones. However, Lysine (K) and Glutamic acid (E) were much abundant in non-malonylation peptides. Thus, we believe that the difference between the two peptides could be a new method to distinguish them.

## Conclusions

In this study, a novel method entitled Mal-Prec was developed to predict human malonylation sites. The best prediction performance was achieved when using PCA to reduce the dimensionality of feature combination (CKSAAP, AAindex, and one-hot) to 100, rather than combined those features all together. By individual comparison of three features (CKSAAP, AAindex, and One-hot), we found that CKSAAP with the incorporation of the first four features, performed best. While the AAindex and one-hot combination performed best in two features combination. This indicated that simply incorporating more features may not achieve the best results. Based on the results obtained by 5-fold cross-validation, Mal-Prec remarkably outperforms existing predictors and could serve as a useful tool for identifying and discovering novel malonylation sites in human proteins. In addition, although good performance has been obtained by using Mal-Prec, there is still space for the method to be refined. First of all, more peptide features, such as structure properties, evolutionary information, and so on, could be incorporated for the prediction. In future, we will take more feature constructions into account to achieve better prediction performance. Secondly, we have not solved the data set imbalance problem. Down-sampling method is popular but not good enough for data set imbalance. We will introduce other approaches to solve the imbalance problem, such as one-side selection (OSS) and sampling based on clustering (SBC), etc. Finally, we are planning to develop a webserver for the method, by doing which other researchers could try this novel method for malonylation site predictions.

## Methods

### Data collection and preprocessing

In this study, the data sets were retrieved from literature [10, 13]. A total of 1768 sequence fragments from 934 human proteins were collected. To reduce the redundancy and avoid artificial bias, CD-HIT was employed to remove redundant sequences with equal to or more than 40% similarities [18]. Then the processed sequences were truncated into 17-residue long sequence segments with lysine (K) located at the center. Each of peptide fragment was defined as follows:

1 $$ \mathrm{P}={\mathrm{R}}_{-\mathrm{n}}{\mathrm{R}}_{-\mathrm{n}+1}\dots {\mathrm{R}}_{-1}\mathrm{K}{\mathrm{R}}_1{\mathrm{R}}_2\dots {\mathrm{R}}_{\upvarepsilon} $$

Where R_ε_ represents the th ε-th downstream peptide from the center K while R_-n_ represents the n-th upstream sequence fragment, and so forth. The length of the sequence fragment is n + ε + 1. Since there might be fewer amino acids around the center K, as shown in Supplementary Figure 3, the downstream peptide form the center K is less than ε, so we can use X to fill in those residues. Thus, the dataset was made up of 20 native amino acids and the dummy code X. Different studies may select varied length of malonylation peptide segments for analysis. In this project, we set ε = 8 and *n* = 8 and the length of the peptide segment is 17. Thus, the complete sequence segment P describing a lysine belongs to either of two classes (δ_1_, δ_2_). If the represented lysine is a malonylation site, then δ_1_ =0, otherwise δ_2_ =1.

2 $$ \mathrm{P}\in {\left({\updelta}_1,{\updelta}_2\right)}^{\mathrm{T}}\ {\updelta}_1,{\updelta}_2\in {\left(0,1\right)}^{\mathrm{T}} $$

Accordingly, 1735 sequence fragments from 931 human proteins were selected as positive dataset. Sequence fragments around lysine (abbreviated as Lys or K) that are not included in the positive data set were constituted as negative dataset. After doing all of this, we obtained 45,607 negative samples. Unbalanced dataset may lead to false prediction, hence we used the down-sampling method to construct a balanced dataset [19]. Therefore, our data set is balanced which contains 3470 sets of data, half of the positive and negative sets. In order to validate the performance of the predictor, we split 20% of the dataset (695) as independent dataset, the remaining are training dataset (2775).

### Flowchart of the proposed method

**Fig. 6:**
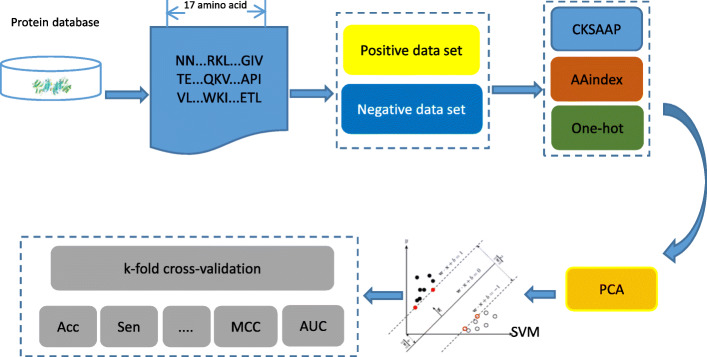
Schematic illustration of the Mal-Prec method from protein data selection to k-fold cross-validation

Flowchart of the malonylation site prediction method Mal-Prec proposed in this paper is shown in Fig. 6. The prediction steps of the Mal-Prec are described as follows:

1. Data collection and preprocessing. Dataset was collected through literature and NCBI websites. Sliding window was then used to select a peptide having a length of 17 with lysine at the center point. Positive data set and negative data set were constructed with equal quantity by down-sampling method.
2. Feature representation. CKSAAP, AAindex, and One-hot coding method were chosen as features to represent each peptide segment in this study.
3. Dimensionality reduction. High dimensional data set may lead to the curse of dimensionality [19]. To solve this problem, we used PCA for dimensionality reduction, and also analyzed the suitable dimension of the data set.
4. Classification. Different data requires corresponding algorithms [20]. Comparing to other classical classifier algorithms, we chose SVM as classification algorithm in Mal-Prec.
5. Model performance evaluation. To find the suitable parameters and avoid potential over-fitting issue, we adopted the 5-fold cross-validation algorithm and employed classical metrics, such as Acc and Sen, etc., to assess the performance of the algorithm.

### Feature construction

#### Binary encoding (one-hot encoding)

Binary encoding is also called one-hot encoding, which could transform amino acids into orthogonal numeric vectors, and has been applied in many protein sequence analyses. Since there are 21 types of amino acids (20 conventional amino acids and 1 pseudo amino acid X), each peptide sequence can be represented as a 21-dimensional vector. For example, the protein sequence is ‘ACDEFGHIKLMNPQRSTVWYX’. Thus, alanine (A) is encoded as ‘100000000000000000000’. In particular, the pseudo amino acid X is encoded as “000000000000000000001”. Suppose the peptide sequence is ‘VAERAALEKLDANQEYK’, we obtained a 17*21-dimensional vector for the peptide after encoding (Supplementary Figure 4).

### Physiochemical properties (AAindex)

AAindex is a database of numerical indices representing various physicochemical and biochemical properties of amino acids. AAindex (release 9.2) consists of three sections: (1) AAindex1 including 566 properties for the amino acid index of 20 numerical values; (2) AAindex2 containing amino acid mutation matrix, and (3) AAindex3 with protein contact potentials. The database could be found at the following URL address https://www.genome.jp/aaindex/. In this paper, nine physical and chemical properties are used, which are hydrophilicity value, mean polarity, isoelectric point, refractivity, average flexibility indices, average volume of buried residue, electron-ion interaction potential values, transfer free energy to surface, and consensus normalized hydrophobicity. The length of each peptide is 17, so the physiochemical properties is 17*9-dimensional vector. The physical and chemical properties are shown in Table 5 below.

**Table 5:** Nine physicochemical properties used in this study

Properties description	Reference
Hydrophilicity value	Hopp and Woods [21]
Mean polarity	Radzicka and Wolfenden [22]
Isoelectric point	Zimmerman et al. [23]
Refractivity	Treece et al. [24]
Average flexibility indices	Bhaskaran and Ponnuswamy [25]
Average volume of buries residue	Chothia [26]
Electron-ion interaction potential values	Cosic [27]
Transfer free energy to surface	Bull and Breese [28]
Consensus normalized hydrophobicity	Eisenberg [29]

### Composition of K-spaced amino acid pairs (CKSAAP)

CKSAAP reflects the composition of K-spaced amino acid pairs that have been successfully applied in many PTM predictions with a competitive performance [30–35]. CKSAAP counts the occurrence frequencies of the k-spaced amino acid pairs in a peptide sequence. For details, because there are 20 types of amino acids and 1 pseudo amino acid X, 21*21 = 441 amino acid pairs could be formed. After we extracted the amino acid pairs separated by K (K = 0, 1, 2, ...) amino acids, we could count the probability that these residues will appear in this 441 amino acid pairs. Hence the generation of a 441-dimensional feature vector. Take the peptide ‘VAERAALEKLDANQEYK’ as an example. With the length set to 17, when k = 0, 17 amino acid pairs {VA, AE, ER, ..., YK,KX} could be extracted, that is, each amino acid and its next adjacent amino acid are combined to form a pair. Therefore, we use N_VA_ counts occurrences of VA, which is recorded as:

3 $$ {N}_{VA}= occurrences\ \left(\mathrm{VA}\right) $$

Then we count the probability that these residues will appear in 441 amino acid pairs.

4 $$ {\left({N}_{VA},{N}_{AE},{N}_{ER},\dots \dots .\right)}_{441} $$

### Operation algorithm

#### Support vector machine

Support Vector Machine (SVM) is a classical supervised classifier based on VC (Vapnik-Chervonenkis) dimensional theory and structural risk minimization principle [36]. It has good generalization ability. The principle of SVM is to map the samples of the input space to the high-dimensional feature space through the kernel function, so as to obtain the optimal classification hyper-plane of the lower VC dimension in the high-dimensional kernel space. It has achieved good performance in many fields, such as protein-protein interaction, protein secondary structure prediction, cancer classification and subtyping, biomarker/Signature discovery, drug discovery for cancer therapy, cancer driver gene discovery, and so on [37–39]. In this paper, SVM is adopted as a classifier.

#### Principal component analysis

PCA (Principal Component Analysis) is a commonly used data analysis method [40]. It is often used for dimensionality reduction of high-dimensional data because transformation of the original data into a set of linearly independent representations could then be used to extract the main feature components of the data [40–42].

### Performance measures

We employed 5-fold cross-validation to conduct model selection, which can effectively avoid over-learning and under-learning and the result was also more persuasive [43]. In 5-fold cross-validation, the whole training data set was divided into 5 subsets with roughly equal size randomly, each subset is in turn taken as test set and the remaining 4 subsets are used to train the classifier. In addition, in order to provide a more intuitive and easier-to-understand method to measure the prediction quality, the following set of five metrics have been used to evaluate the prediction performance, which are Accuracy (Acc), Sensitivity (Sen), Specificity (Spec), F1 score, and Matthews correlation coefficient (MCC). The selected performances have been demonstrated in eqs. (5)–(9).

5 $$ \mathrm{Acc}=\frac{\mathrm{TP}+\mathrm{TN}}{\mathrm{TP}+\mathrm{FP}+\mathrm{TN}+\mathrm{FN}} $$

6 $$ \mathrm{Sen}=\frac{\mathrm{TP}}{\mathrm{TP}+\mathrm{FN}} $$

7 $$ \mathrm{Spec}=\frac{TN}{TN+ FP} $$

8 $$ \mathrm{F}1=2\times \frac{SN\times PPV}{SN+ PPV} $$

9 $$ \mathrm{MCC}=\frac{\left(\mathrm{TP}\times \mathrm{TN}\right)-\left(\mathrm{FP}\times \mathrm{FN}\right)}{\sqrt{\left(\mathrm{TP}+\mathrm{FN}\right)\times \left(\mathrm{TN}+\mathrm{FP}\right)\times \left(\mathrm{TP}+\mathrm{FP}\right)\times \left(\mathrm{TN}+\mathrm{FN}\right)}} $$

Where TP, TN, FP and FN represent the numbers of true positives, true negatives, false positives and false negatives, respectively [44]. In addition, the receiver operating characteristic (ROC) curves are plotted based on Sen and Spec by taking different thresholds [45] and their area under the ROC (AUC) values were also calculated based on the trapezoidal approximation [46].

## Supplementary Information

**Additional file 1: Table S1.** The performance of the proposed method using different CKSAAP features. **Table S2.** Performance of the proposed method using different CKSAAP combinations. **Table S3.** The performance of 5-fold cross-validation (dimensions equal to 100). **Table S4.** The performance of 5-fold cross-validation without PCA. **Table S5.** The performance comparison of different single feature. **Table S6.** The performance comparison of different feature combination.

**Additional file 2: Figure S1.** Comparison of accuracy in different dimensions got by using PCA. **Figure S2.** The comparison of different feature combinations. **Figure S3.** Schematic diagram of malonylation sequence fragment. X represents filled-up residues in the fragment. n and ε represent the n-th upstream peptide and ε-th downstream peptide from the center K, respectively. **Figure S4.** Transformation of the 17-amino-acid peptide VAERAALEKLDANQEYK into a 17*21 dimensional vector after one-hot encoding process.

## Data Availability

All data generated or analysed during this study are included in this published article and its supplementary information files.

## Abbreviations

Mal-Prec: Malonylation Prediction
PTM: Post-Translational Modification
T2DM: Type 2 Diabetes Mellitus
PCA: Principal Component Analysis
SVM: Support Vector Machine
AAindex: Amino Acid Index Properties
CKSAAP: Composition of K-Spaced Amino Acid Pairs
