# Supplementary material for: Mal-Prec: computational prediction of protein Malonylation sites via machine learning based feature integration: Malonylation site prediction
Source: BMC Genomics. 2020 Nov 23;21:812. doi: 10.1186/s12864-020-07166-w (PMC7682087; doi:10.1186/s12864-020-07166-w)
Supplement: Supplementary file 1 — Additional file 1: Table S1. The performance of the proposed method using different CKSAAP features. Table S2. Performance of the proposed method using different CKSAAP combinations. Table S3. The performance of 5-fold cross-validation (dimensions equal to 100). Table S4. The performance of 5-fold cross-validation without PCA. Table S5. The performance comparison of different single feature. Table S6. The performance comparison of different feature combination. [file 12864_2020_7166_MOESM1_ESM.docx]

**Supplementary Table 1** The performance of the proposed method using different CKSAAP features.

| **K** | **Acc (%)** | **Sen (%)** | **Spec (%)** | **F1 (%)** | **MCC (%)** |
| --- | --- | --- | --- | --- | --- |
| 0 | 82.63 | 86.78 | 78.21 | 83.62 | 71.09 |
| 1 | **82.85** | 84.07 | 81.69 | 83.00 | **71.48** |
| 2 | 81.59 | 87.19 | 75.92 | 82.66 | 69.76 |
| 3 | 82.02 | 85.45 | 78.43 | 82.68 | 70.33 |
| 4 | 82.74 | 82.84 | 82.72 | 82.71 | 71.36 |
| 5 | 82.70 | 83.32 | 82.08 | 82.77 | 71.11 |
| 6 | 82.63 | 83.11 | 82.20 | 82.49 | 71.11 |
| 0+1 | 82.05 | 81.86 | **82.24** | 82.02 | 70.29 |
| 0+1+2 | 81.66 | 81.46 | 81.37 | 81.49 | 69.48 |
| 0+...+3 | 82.67 | 89.68 | 75.36 | 83.63 | 70.59 |
| 0+...+4 | 82.56 | 95.88 | 69.02 | 84.58 | 70.15 |
| 0+...+5 | 82.41 | 98.23 | 66.54 | 84.70 | 69.50 |
| 0+...+6 | 82.23 | **98.63** | 65.72 | **84.77** | 69.19 |
| mean | 82.36±0.42 | 87.58±6.17 | 77.04±6.20 | 83.16±1.03 | 70.42±0.78 |

**Supplementary Table 2** Performance of the proposed method using different CKSAAP combinations

| **K** | **Acc (%)** | **Sen (%)** | **Spec (%)** | **F1 (%)** | **MCC (%)** |
| --- | --- | --- | --- | --- | --- |
| 0 | 88.76 | 89.92 | 87.53 | 89.11 | 80.01 |
| 1 | 88.94 | 90.27 | 87.64 | 89.19 | 80.30 |
| 2 | 89.30 | 89.79 | 88.78 | 89.42 | 80.89 |
| 3 | 89.87 | 89.99 | 89.77 | 89.87 | 81.81 |
| 4 | 88.58 | 89.01 | 88.16 | 88.65 | 79.79 |
| 5 | 89.37 | 90.09 | 88.68 | 89.40 | 81.00 |
| 6 | 89.41 | 90.38 | 88.31 | 89.58 | 81.02 |
| 0+1 | 89.19 | 89.58 | 88.84 | 89.22 | 80.73 |
| 0+1+2 | 89.91 | 90.46 | 89.31 | 89.93 | 81.87 |
| 0+…+3 | **91.24** | **91.71** | **90.83** | **91.18** | **84.03** |
| 0+...+4 | 90.63 | 91.41 | 89.97 | 90.63 | 83.02 |
| 0+...+5 | 90.49 | 91.41 | 89.50 | 90.64 | 82.76 |
| 0+...+6 | 89.44 | 91.25 | 87.64 | 89.60 | 81.12 |

**Supplementary Table 3** The performance of 5-fold cross-validation (dimensions equal to 100)

| **K** | **Acc (%)** | **Sen (%)** | **Spec (%)** | **F1 (%)** | **MCC (%)** |
| --- | --- | --- | --- | --- | --- |
| 1 | 92.97 | 94.68 | 91.21 | 93.19 | 86.92 |
| 2 | 89.55 | 90.91 | 88.32 | 89.22 | 81.26 |
| 3 | 90.63 | 89.47 | 91.85 | 90.75 | 83.01 |
| 4 | 91.53 | 94.03 | 89.20 | 91.47 | 84.49 |
| 5 | 91.53 | 89.45 | 93.57 | 91.28 | 84.48 |
| mean | 91.24±1.24 | 91.71±2.50 | 90.83±2.10 | 91.18±1.43 | 84.03±2.09 |

**Supplementary Table 4** The performance of 5-fold cross-validation without PCA

| K | **Acc (%)** | **Sen (%)** | **Spec (%)** | **F1 (%)** | **MCC (%)** |
| --- | --- | --- | --- | --- | --- |
| 1 | 74.59 | 64.60 | 85.61 | 72.73 | 61.40 |
| 2 | 70.63 | 61.85 | 78.95 | 67.20 | 57.81 |
| 3 | 72.43 | 62.22 | 82.11 | 68.71 | 59.16 |
| 4 | 74.23 | 67.13 | 81.78 | 72.87 | 61.40 |
| 5 | 75.68 | 66.04 | 84.67 | 72.39 | 62.40 |
| mean | 73.51±1.99 | 64.37±2.32 | 82.62±2.63 | 70.78±2.64 | 60.43±1.89 |

**Supplementary Table 5** The performance comparison of different single feature.

| **Feature** | **Acc (%)** | **Sen (%)** | **Spec (%)** | **F1 (%)** | **MCC (%)** |
| --- | --- | --- | --- | --- | --- |
| CKSAAP | **82.38** | **91.66** | **74.16** | **83.69** | **69.83** |
| AAindex | 59.78 | 63.87 | 55.81 | 61.27 | 51.76 |
| One-hot | 59.75 | 65.52 | 53.86 | 62.14 | 51.62 |

**Supplementary Table 6** The performance comparison of different feature combination.

| **Features** | **Acc (%)** | **Sen (%)** | **Spec (%)** | **F1 (%)** | **MCC (%)** |
| --- | --- | --- | --- | --- | --- |
| CKSAAP (exclude) | 85.44 | 85.82 | 85.14 | 85.52 | 75.09 |
| AAindex (exclude) | 78.27 | 79.81 | 76.65 | 78.66 | 65.98 |
| One-hot (exclude) | 69.77 | 73.61 | 65.92 | 70.96 | 57.64 |
| ALL | **91.24** | **91.71** | **90.83** | **91.18** | **84.03** |
